# Salivary biochemical parameters in people living with HIV on ART and dental caries: a cross-sectional study in Monastir, Tunisia

**DOI:** 10.1186/s12903-023-03821-2

**Published:** 2024-01-06

**Authors:** Latifa Berrezouga, Ikbel Kooli, Wafa Marrakchi, Fadoua Neffati, Fadhel Najjar, Mohamed Chakroun

**Affiliations:** 1 Department of Microbiology and Immunology, Faculty of Dental Medicine, University of Monastir, Monastir, Tunisia; 2 Department of Endodontics, Dental Clinic, University of Monastir, Monastir, Tunisia; 3 Laboratory of Medical and Molecular Parasitology and Mycology LR12ES09, Faculty of Pharmacy, University of Monastir, Monastir, Tunisia; 4 Department of Infectious Diseases, F. Bourguiba Teaching Hospital, University of Monastir, Monastir, Tunisia; 5 Department of Biochemistry and Toxicology, F. Bourguiba Teaching Hospital, University of Monastir, Monastir, Tunisia

**Keywords:** HIV/AIDS, Antiretroviral Therapy, Saliva, Dental Caries, Cross-sectional Study, Tunisia

## Abstract

**Background:**

Studies regarding salivary biochemical parameters and dental caries in adult people living with HIV/AIDS (PLWHA) are scanty. *Aim*: To investigate salivary biochemical parameters and dental caries in adult PLWHA who are on antiretroviral therapy (ART) and compare the findings with people negative for HIV infection.

**Methods:**

The study included 50 HIV positive individuals as a test group (TG) and 50 HIV negative individuals as a control group (CG). Dental examination was performed according to WHO guidelines to assess DMFT. Digital panoramic radiographs were taken to detect additional infectious foci. Non-stimulated saliva was collected between 9 and 12 a. m for 5 min to evaluate 18 biochemical parameters and salivary flow rate (SFR). Parametric and non parametric tests were used according to data distribution. The level of significance was set at *p *< 0.05%.

**Results:**

Patients’ mean ages and M/F sex ratios for TG and CG were 38.80 ± 9.69 y/o. vs. 37.98 ± 13.47 y/o. and 3.54 vs. 2.33, respectively. Higher means of decayed teeth were recorded in TG, 4.47 ± 3.00 vs. 3.88 ± 2.81 in CG with no significant difference (*p* = 0.41). Means of filled teeth were significantly lower in TG 2.38 ± 2.16 vs. 4.16 ± 3.35 in CG (*p* = 0.01), respectively. No statistical significant difference was noted in DMFT indices between the 2 groups (8.04 ± 6.90 vs. 8.52 ± 6.24, *p* = 0.71). The following salivary parameters were significantly lower in TG compared to CG, respectively: mean SFR 0.44 ± 0.18 ml/min vs. 0.61 ± 0.26 ml/min; median levels of sodium and chlorides, 4 mmol/L and 13.5 mmol/L vs. 9 mmol/L and 19 mmol/L (*p* < 0.001) and uric acid, 103.50 mmol/L vs. 163 (*p* = 0.009). However, higher median levels were recorded with calcium, 1.09 mmol/L vs. 0.54 (*p* < 0.001) and sIgA 23 mg/dl vs. 5 mg/dl (*p* < 0.001). In TG, a positive correlation was found between DC, potassium, urea, and chlorides (*p* < 0.05). Salivary renal and hepatic biomarkers were comparable between the two groups.

**Conclusions:**

PLWHA have shown an alteration in some salivary parameters, more decayed teeth and less filled teeth. Preventive measures should be implemented to lower dental caries and enhance accessibility to oral care services. In addition, saliva can be utilized to monitor oral and general health status among PLWHA on ART.

## Background

HIV/AIDS epidemic remains one of the most serious affections worldwide. According to the UNAIDS’ recent report, putting an end to the disease by 2030 is still in danger [1]. In fact, many countries are far from the 95–95-95 UNAIDS and WHO prevention program targets [2]. In 2022, an estimated 39 million of people are living with HIV with an increase in frequency and severity in Sub-Saharan Africa and Asia along with an estimated 630.000 HIV-related deaths. Indeed, an estimated 29.8 million of people are accessing antiretroviral treatment (ART) [2]. The epidemic is on the rise with annually new HIV infections in the MENA region (Middle East and North Africa), Eastern Europe, Central Asia and Latin America. In the MENA region, the reported number of PLWHA is 190.000 (160.000–220.000) and only 88.000 are accessing ART [2–4]. In Tunisia, although low (< 0.1 [< 0.1–0.1]), the prevalence rate of HIV/AIDS is increasing. The estimated number of PLWHA is 7.100 (5200–10.000) [5].

Despite a marked decline in HIV-related mortality in the ART era and the shift from a life-threatening disease to a chronic one, PLWHA are still suffering from HIV-related complications, co-morbidities and ART-related side effects [6–8]. Several determinants that negatively affect people’s quality of life have been reported [9–11]. In the oral cavity, saliva with its immune and non-immune components is a key protective barrier against diseases. It is composed of water (99%), proteins (immunoglobulins, mucins, albumin), electrolytes (sodium, potassium, phosphate, chlorine, bicarbonate, urea, magnesium) and enzymes (alkaline phosphatase, lactate desydrogenase, amylase), in addition to its anti-oxidative properties (uric acid, superoxide dismutase enzyme). Therefore, the impairment of saliva composition by HIV infection, ART regimen and poor oral hygiene may predispose the patient to oral diseases. Affections like candidiasis, dental caries (DC), periodontal diseases, hyperpigmentation and salivary glands dysfunction are still common [12–16]. A part from cariogenic bacteria such as *S. mutans* and lactobacilli, several contributing factors are reported to be involved in DC like age, ART duration, carbohydrate-rich diet, tobacco and drugs use [17].

It should be noted that the literature search identified two types of studies: those that deal with DC and HIV-related risk factors and others that focused on salivary components in individuals who are HIV-infected. Almost no publications have been reported about salivary biochemical parameters and DC in PLWHA. Regarding DC in PLWHA, high DMFT indices have been commonly recorded [18, 19]. Mean DMFT indices of 2.28 ± 3.68 were noted in HIV positive people compared to HIV negative individuals (1.29 ± 2.21) [20]. ART duration has been linked to an increase in DC risk, apart from the risk factors that have been reported for DC [19]. However, some authors failed to recognize ART as a risk factor [21, 22]. Indeed, there were conflicting results about how HIV and/or ART affect saliva parameters. Patients with symptomatic conditions or those receiving ART had lower salivary flow rates (SFRs) and alteration of saliva components [13, 23, 24]. Unlike previous studies, ART has been reported to have beneficial effects on the immune system, SFR, and any salivary components, including IgA, calcium, magnesium, total proteins, LDH, and anti-oxidative agents [16].

Based on the aforementioned information, the objective of the present research was i: to investigate salivary biochemical parameters and DC in adult PLWHA on ART ii: to compare the findings with people negative for HIV infection and iii: to investigate the associations between DC and salivary parameters. The following hypothesis were considered: the null hypothesis (H0), assumes that salivary parameters and DMFT indices in PLWHA receiving ART are comparable to those of HIV negative people, and an alternative hypothesis (H1), assumes that salivary parameters and DMFT indices in PLWHA receiving ART are not comparable to those of HIV negative people.

## Methods

### Study type and location

This cross-sectional study, conducted according to STROBE checklist (strengthening the reporting of observational studies in epidemiology) [25] was performed at the department of infectious diseases of F. Bourguiba teaching hospital, the department of restorative dentistry and endodontics for oral examination and saliva sample collection, and the laboratory of biochemistry and toxicology for biochemical analysis, Monastir, Tunisia. One hundred and fifty PLWHA are treated and followed in a day hospital by specialists in infectious diseases. The team includes nurses for periodical biological monitoring, a pharmacist for ART delivery, a psychologist and a social assistant for counseling support along with specialists.

### Ethical consideration

The present study was approved by the committee of ethics of the faculty of pharmacy, Monastir, Tunisia, under the reference CER-SVS/ISBM 013/2020, and is part of Latifa Berrezouga. IADR research project on PLWHA. The study was conducted with respect to Helsinki Declaration. Informed consent was obtained from all participants.

### Data collection

#### Inclusion and exclusion criteria

For the test group (TG), individuals who were already scheduled for a routine clinical and biological follow-up and ART pick-up were included based on the following criteria: men and women aged 18 years and older and had at least 15 teeth present in the oral cavity. For the control group (CG), patients were matched for age and gender. They were healthy, negative for HIV infection and had at least 15 teeth present in the oral cavity. The following participants were excluded from the study: children and pregnant women, non-consenting people and those with HIV-related and non-related oral and systemic diseases (Table 1).

**Table 1:** Inclusion and exclusion criteria for Test group (*n* = 50) and Control group (*n* = 50)

	Inclusion criteria	Exclusion criteria
People living with HIV receiving ART (TG)	-Men and women. -Age ≥ 18 years old. -At least 15 teeth present in the oral cavity.	-Symptomatic oral lesions. -HIV and/or ART-related Co-morbidities (e.g. cardiac, renal, and hepatic). -Children, pregnant women. -Edentulous patients. -Non-consenting patients.
Control group	-Negative for HIV infection. -Age and gender matched. -At least 15 teeth present in the oral cavity.	-HIV-related risk factors. -Symptomatic oral lesions. -Systemic diseases (e.g. cardiac, renal, and hepatic). -Medications. -Children, pregnant women. -Edentulous patients. -Non-consenting patients.

### Sample size calculation

A sample size of 44 participants was identified using the Krejcie and Morgan table [26] based on an average of 50 patients attending the department of infectious diseases per month, assuming a population proportion of 0.5% and a confidence interval of 95%. A final sample of 50 participants was considered. Data collection was performed from 8.30 a. m to 2 p. m, 3 days per week over a period of 6 months, from Mai 2022 to October 2022.

### Oral examination and DMFT assessment

Demographic and personal data (age, gender, health status, medication, phone number, origin, dental status) of all patients were recorded on a clinical sheet. Dental examination was performed by an expert dentist (L.B) using a plane mirror and a periodontal probe. Assessment of decayed and missing teeth due to caries, and filled and sound teeth (including third molars) was done according to WHO guidelines [27].

### Digital panoramic radiographs

Panoramic radiographs were taken using Scanora 3D device (Scoredex, Finland) according to manufacturer’s instructions for adults (73 kV, 8 mA, 15 s, X 50 mm and Y-38 mm) to detect hidden caries, root caries and caries under prosthetic restorations.

### Saliva collection

Unstimulated saliva was collected between 9 and 12 a. m, using the spitting method [28]. Subjects were previously instructed not to eat, drink or brush their teeth at least 2 h before the procedure. Patients were invited to rinse the oral cavity with distilled water, then to lean forward and spit the saliva into a sterile graduated tube for 5 min. The salivary flow rate (ml/min) was immediately evaluated. All samples were centrifuged at 3500 rpm at 4°C to eliminate debris and viscosity and the supernants were stored in eppendorf tubes at -20°C.

### Biochemical analysis

It's noteworthy that the parameters tested are the same ones used for routine blood test monitoring of PLWHA on ART in the department of infectious diseases. Thus, saliva was utilized to investigate these parameters. Analysis was performed with COBAS 6000 TM autoanalyzer (Roche Diagnostic, Mannheim, Germany). The following methods were used: Enzymatic for urea and creatinine; Potentiometric for chloride (Cl), potassium (K) and sodium (Na); Colorimetric for calcium (Ca) and total proteins; Turbidimetric for sIgA (salivary Immunoglobulin A) and albumin; Molybdate UV for phosphorus (P) and by Kinetic method for gamma glucosyltransferase (γ GT), alkalin phosphatase (AP), amylase (405 nm), aspartate aminotransferase (AST), alanine aminotransferase (ALT), lactate deshydrogenase (LDH) and creatine phosphokinase (CPK) (340 nm). Urea and creatinine are renal markers. γ GT, AST, ALT, LDH and CPK are hepatic markers. All samples were tested in duplicate.

### Statistical analysis

Data analysis was performed using SPSS vs. 21.0 software (Statistical Package for Social Sciences, USA). Kolmogorov-Smimov test was used to assess distribution of variables. In case of Gaussian distribution, the Student t-test for independent samples was used to compare means (± standard deviation) between groups; otherwise, the non-parametric U test of Mann–Whitney was used for comparison of medians (min–max). Correlations between continuous variables were analyzed using the Spearman’s test. The result was considered statistically significant with *p* < 0.05.

## Results

Patients’ mean ages and M/F sex ratios for TG and CG were 38.80 ± 9.69 y.o vs. 37.98 ± 13.47 y.o and 3.54 vs. 2.33, respectively. All PLWHA were under highly active ART with a mean duration of 6.68 ± 6.4 years. The mean level of serum CD4 T lymphocytes was 613.42 ± 254.63. Nearly 65% (64.6%) of the patients had CD4 counts ≥ 500 cells/mm^3^ and 88.9% had CD4 counts ≥ 350 cells/mm^3^. Only two patients had CD4 counts of 45 and 175 cells/mm^3^, respectively. The RNA-viral load was undetectable in 81.6% of the cases (< 20 copies/mm^3^).

The total number of teeth was 1419 in TG vs. 1416 teeth in CG (*p* > 0.05). Table 2 shows higher means of decayed teeth in TG 4.47 ± 3.00 vs. 3.88 ± 2.81 in CG, with no statistical significant difference (*p* = 0.41). Means of filled teeth were significantly lower in TG, 2.38 ± 2.16 vs. 4.16 ± 3.35 (*p* = 0.01). Comparable means were observed between the 2 groups in missing teeth. DMFT indices between TG (8.04 ± 6.90) and CG (8.52 ± 6.24) were comparable (*p* = 0.71).

**Table 2:** Means (± SD) of decayed, missing, filled teeth, DMFT indices in Test group (*n* = 50) and in Control group (*n* = 50)

Teeth/age	Test Group	Control group	*p*. value
Age	38.80 ± 9.69	37.98 ± 13.47	0.72
Decayed	4.47 ± 3.00	3.88 ± 2.81	0.41
Missing	5.66 ± 5.27	5.72 ± 7.56	0.97
Filled	2.38 ± 2.16	4.16 ± 3.35	0.01*
DMFT	8.04 ± 6.90	8.52 ± 6.24	0.71

^*^Significant

In TG, the results of Table 3 revealed more decayed teeth and less filled teeth in the posterior sector 6.30 ± 2.10 and 2.60 ± 2.34, respectively. A strong significant difference was recorded in the filled component between TG and CG (*p* = 0.009).

**Table 3:** Means (± SD) of decayed, missing and filled teeth by sector in Test group (*n* = 50) and in Control group (*n* = 50)

Sector	Test group	Control group	*p*. value
**Anterior**			
Decayed	1.41 ± 1.24	1.66 ± 1.30	0.63
Missing	1.50 ± 1.56	1.16 ± 1.02	0.54
Filled	2 ± 1.85	2.25 ± 1.42	0.71
**Posterior**			
Decayed	6.30 ± 2.10	5.20 ± 2.64	0.15
Missing	8.15 ± 5.15	8.45 ± 8.46	0.89
Filled	2.60 ± 2.34	5.30 ± 3.67	0.009*

^*^Strongly significant

Concerning SFR, significantly lower means were found in TG compared to CG, 0.44 ± 0.18 vs. 0.61 ± 0.26, respectively (*p* < 0.001). Moreover, in TG, an SFR < 0.3 ml/min or between 0.3–0.4 ml/min was recorded in 48% and 34%, respectively. However, most patients in CG (70%) had an SFR > 0.4 ml/min (Table 4).

**Table 4:** Saliva flow rates (SFR) in Test group (*n* = 50) and in Control group (*n* = 50)

Salivary flow rate	Test group (mean ± SD)	Control (mean ± SD)	*p*. value
Total SFR (5 min)	2.19 ± 0.91	3.08 ± 1.32	< 0.001*
SFR (ml/min)	0.44 ± 0.18	0.61 ± 0.26	< 0.001*
	^a^N and ^b^%	N and %	
SFR < 0.3 ml/min	9–18	2–4	**–**
SFR 0.3–0.4 ml/min	24–48	13–26	**–**
SFR > 0.4 ml/min	17–34	34–70	**–**

^*^Strongly significant

^a^N: number

^b^%: percentage

Table 5 depicts results of electrolytes, proteins and enzymes. In TG, significant lower median levels of Na, Cl and uric acid were noted, 4 mmol/L, 13.5 mmol/L and 103.50 mmol/L, respectively (*p* = 0.000). However higher median levels were recorded for Ca, 1.09 mmol/L (*p* = 0.000) and for sIgA, 23 mg/dl (*p* = 0.000).

**Table 5:** Distribution of salivary biochemical parameters in Test group (*n* = 50) and in Control group (*n* = 50) (means and medians)

Parameters	Test Group	Control	*p*. value
Creatinine (mmol/L) (min–max)	7 (5–27)	6 (2–18)	0.41
Urea (mmol/L)	6.856 ± 3.67	6.62 ± 2.69	0.72
Uric acid (mmol/L) (min–max)	103.50 (3–930)	163 (3–622)	0.009*
Sodium (mmol/L) (min–max)	4 (8–23)	9 (8–33)	0.000*
Potassium (mmol/L)	22.12 ± 5.28	24.15 ± 7.10	0.10
Chlorides (mmol/L) (min–max)	13.5 (6–24)	19 (11–46)	0.000*
Calcium (mmol/L) (min–max)	1.09 (0.80–2.50)	0.54 (0.10–5.73)	0.000*
Phosphorus (mmol/L)	6.26 ± 3	5.81 ± 2.44	0.41
Total proteins (g/L)	0.70 ± 0.33	0.68 ± 0.39	0.81
Albumin (g/L)	99.59 ± 72.95	129.99 ± 112.85	0.11
sIgA (mg/dl) (min–max)	23 (0.10–73)	5 (0.10–92)	0.000*
Amylase (UI/L) (min–max)	139.332 (17.400–2795.000)	133.850 (31.900–356.300)	0.54
Alkalin phosphatase (UI/L) (min–max)	7 (5–32)	5 (5–76)	0.87
AST (UI/L) (min–max)	29.50 (5–304)	27.0 (1–447)	0.54
ALT (UI/L) (min–max)	12.50 (5–199)	6.50 (5–397)	0.42
LDH (UI/L) (min–max)	325 (10–1348)	222.500 (10–968)	0.31
CPK (UI/L) (min–max)	12.5 (7–46)	10 (1–252)	0.84
γ GT (UI/L)	9.10 ± 5.74	7.96 ± 5.49	0.31

*Abbreviation*: *sIgA* salivary immunoglobulin A, *AST* aspartate aminotransferase, *ALT* alanine aminotransferase, *LDH* lactate deshydrogenase, *CPK* creatine phosphokinase, *γ GT* gamma glucosyltransferase

^*^Strongly significant

The Spearman test was used to investigate the correlation between DMFT indices and salivary parameters. In TG, DMFT indices were positively associated to K (*r* = 0.30, *p* = 0.031), urea (*r* = 0.30, *p* = 0.033) and Cl (*r* = 0.31, *p* = 0.026). In CG, DMFT indices were negatively associated to K (*r* = -0.30, *p* = 0.034) and uric acid (*r* = -0.30, *p* = 0.029) and positively associated to age (*r* = 0.40, *p* = 0.004). No significant association was recorded for the remaining parameters; including ART and serum CD4 (Table 6).

**Table 6:** Correlation between DMFT, age, ART, serum CD4 and salivary parameters in Test group (*n* = 50) and in Control group (*n* = 50)

DMFT/TG	r	*p*. value	DMFT/CG	r	*p*. value
Age	-0.19	0.17	Age	0.404	0.004*
ART	-0.14	0.39	^a^–-	–-	–-
CD4	0.01	0.93	^b^–-	–-	–-
Sodium	0.24	0.09	Sodium	0.08	0.56
Potassium	0.30	0.031*	Potassium	-0.30	0.034*
Urea	0.30	0.033*	Urea	-0.23	0.09
Chlorides	0.31	0.026*	Chlorides	-0.17	0.23
Phosphorus	0.24	0.08	Phosphorus	-0.25	0.08
Calcium	-0.11	0.41	Calcium	0.13	0.34
Amylase	-0.07	0.61	Amylase	-0.18	0.19
Albumin	-0.02	0.89	Albumin	0.03	0.82
Phosphatase alkaline	0.05	0.72	Phosphatase alkaline	0.15	0.28
Total proteins	0.19	0.17	Total proteins	-0.14	0.22
sIgA	0.12	0.38	sIgA	0.15	0.27
Uric acid	0.17	0.22	Uric acid	-0.30	0.029*
Age	-0.19	0.17	Age	0.40	0.004*
SFR	-0.15	0.29	SFR	0.11	0.41

^*^Significant

^a^Not concerned

^b^Not performed

## Discussion

The literature lacks studies on salivary parameters and DC in adult PLWHA. Prevalence of DC and associated risk factors are the main focus of most publications [17–20]. Few studies have addressed salivary parameters in PLWHA [12, 16, 23, 24, 29] and only one study has been reported about sIgA and DC in PLWHA [30]. To the best of our knowledge, this study is the first to investigate 18 biochemical salivary parameters and SFR in adult PLWHA, as well as and DC in PLWHA receiving ART.

As for DC, mean DMFT indices in the studied groups were higher compared to Murererehe J et al. findings, 2.28 ± 3.68 for HIV positive people vs. 1.29 ± 2.21 for unaffected people [20], but lower than what Muralidharan S et al. (10.1) [31] and Kumar S et al. reported (12.83 ± 9.6) [32]. Although not significant, this study found that PLWHA had a higher number of decayed teeth in the posterior sector. Unlike what is reported by some authors [21, 22], no correlation was observed between DC and ART.

Regarding the filled teeth component, PLWHA presented significantly less filled teeth compared to HIV negative individuals. This finding corroborates the results of Arubaku W et al. and refers to inequalities in accessing oral health services [33]. We have, previously, shown in a pilot study, that PLWHA quality of life was affected on the psychological, social and environmental levels [34]. This group of people, victim of stigma and discrimination, carries a higher burden of oral diseases [33, 35].

The present study shows that DC is a common finding in both PLWHA and HIV negative individuals. This might reflect not only a common poor oral hygiene, but also the worsening of oral hygiene in Tunisia in the last ten years. Increase in poverty due to the sharp economic crisis and the inability to access health care services could be amongst the dramatic consequences of the Tunisian revolution (people uprising) along with the negative impact of Covid-19 pandemic [36].

Our findings revealed that HIV positive individuals experience a significant decrease in SFR compared to HIV negative individuals. These results are in line with several reports [13, 23, 29]. Indeed, the benefits of ART in increasing SFR are suggested when we observed that 82% of the studied patients had their SFR (≥ 0.3 ml/min) within the normal range of unstimulated SFR (0.3–0.4 ml/min) [14, 37, 38]. In fact, Cherian AP et al. noted that in symptomatic patients at the AIDS stage SFR was very low (0.11ml/min) compared to asymptomatic patients and patients on ART [23]. However, ART's negative effects cannot be discerned from HIV-related oral disease manifestations, as stated by Nizamudin I et al. Didanosine, as an ART drug, has been linked to subjective xerostomia (dry mouth), and some protease inhibitors have been linked to a significant reduction in stimulated and unstimulated salivation [13]. The virus itself may negatively impacts salivary gland functions, resulting in hyposalivation and even xerostomia. Consequently, the buffer capacity of saliva is impacted by the reduction of bicarbonate concentrations, salivary pH, and salivary immune competency [39].

In the present study, sIgA levels were significantly higher in PLWHA compared to HIV negative people. This could be explained by the immune humoral response to HIV infection and the effect of ART on the immune system restoration, as well (with high levels of CD4 + T lymphocytes). Escalona LA et al. showed significant differences of sIgA in unstimulated saliva between HIV positive and HIV negative people [40], while Shahar E et al. [16] reported that sIgA and other salivary components reverted to normal levels in adult PLWHA patients following ART. It should be noted that IgA is the protein that is most common in mucosal secretions and that sIgA plays a crucial function in immune exclusion. It prevents pathogens, particularly HIV-1/2, from gaining access to epithelial receptors, in addition to their neutralizing properties [41, 42]. Contrary to Mandal PK et al. report [30], we did not find a correlation between sIgA levels and DC.

Our results showed a significant increase in Ca levels and a significant decrease in uric acid, Na and Cl levels in PLWHA. Biocina LD et al., observed an increase in calcium levels with highly active ART [23]. However, for Mahajan PG et al. the increase of calcium levels were due to the virus and the alteration of Na, K and Cl could be due to the combined effect of ART and HIV [12]. In addition, Shahar E et al. showed that electrolytes, including Ca, reverted to normal in people on highly active ART [16]. As for uric acid which is the main antioxidant in saliva, its significant decrease in the studied patients may explain the alteration of oxidative properties of saliva. The study of Ahmadi-Motamayel, F et al. showed a significant decrease in uric acid in HIV infected patients compared to healthy people [43].

The role of salivary electrolytes (Na, K, Ca, P, Cl), immune components (sIgA, total proteins, albumin, enzymes (PK, amylase) urea and uric acid is crucial in preventing DC. The decrease of uric acid, Na and Cl levels in PLWHA might play a role in DC occurrence. A positive correlation was only found between DMFT, urea, K and Cl. The increase of calcium levels might play a role in preventing enamel dissolution by acidogenic bacteria of the oral cavity. Based on these findings, the alternative hypothesis has been confirmed.

### Strengthens and limits of the study

The literature is sparse on salivary biomarkers for adult PLWHA. To the best of our knowledge, this is the first study on adult PLWHA investigating 18 biochemical salivary parameters and SFR along with DC using clinical examination and digital panoramic radiographs. Therefore, our findings were discussed based on the available literature. Regarding limitations, we did not report DC prevalence to prevent bias related to the small sample size. Another limitation with cross-sectional studies is the difficulty to establish a causal inference.

Including HIV patients before and after ART initiation and a healthy control group is recommended in order to determine whether changes in salivary parameters are linked to the virus, ART treatment, or both. Finally, to identify key salivary biomarkers in HIV/AIDS patients with and without ART, a larger sample is required.

## Conclusion

PLWHA have more decayed and significantly less filled teeth than HIV negative individuals have. These findings illustrate inequalities in accessing oral health services, and imply stigma and discrimination suffered by HIV/AIDS people. An alteration of SFR and other salivary components have been recorded, despite the benefits of ART in restoring the immune system functions. Thus, preventive measures should be implemented by policy decision makers; along with health care professionals to enhance oral hygiene, improve the wellbeing of individuals, and foster inclusive governance for health. As saliva collection is safe, simple and non-invasive, routine salivary tests could be used to monitor HIV people oral and general health.

## Data Availability

All data are displayed in the manuscript. They are available and could be sent upon request from the corresponding author.

## Abbreviations

HIV/AIDS: Human immunodeficiency virus/ Acquired immunodeficiency syndrome
PLHIV: People living with HIV
QoL: Quality of life
WHO: World health organisation
ART: Antiretroviral therapy
